# Identification of Two Novel Mutations in the *PHEX* Gene in Chinese Patients with Hypophosphatemic Rickets/Osteomalacia

**DOI:** 10.1371/journal.pone.0097830

**Published:** 2014-05-16

**Authors:** Hua Yue, Jin-bo Yu, Jin-wei He, Zeng Zhang, Wen-zhen Fu, Hao Zhang, Chun Wang, Wei-wei Hu, Jie-mei Gu, Yun-qiu Hu, Miao Li, Yu-juan Liu, Zhen-Lin Zhang

**Affiliations:** 1 Department of Osteoporosis, Metabolic Bone Disease and Genetic Research Unit, Shanghai Jiao Tong University Affiliated Sixth People’s Hospital, Shanghai, P. R. China; 2 Department of pediatrics, Shanghai Jiao Tong University Affiliated Sixth People’s Hospital, Shanghai, P. R. China; 3 Department of Orthopedic Surgery, Shanghai Jiao Tong University Affiliated Sixth People’s Hospital, Shanghai, P.R. China; Odense University hospital, Denmark

## Abstract

**Objective:**

X-linked dominant hypophosphatemia (XLH) is the most prevalent form of inherited rickets/osteomalacia in humans. The aim of this study was to identify *PHEX* gene mutations and describe the clinical features observed in 6 unrelated Chinese families and 3 sporadic patients with hypophosphatemic rickets/osteomalacia.

**Methods:**

For this study, 45 individuals from 9 unrelated families of Chinese Han ethnicity (including 16 patients and 29 normal phenotype subjects), and 250 healthy donors were recruited. All 22 exons and exon-intron boundaries of the *PHEX* gene were amplified by polymerase chain reaction (PCR) and directly sequenced.

**Results:**

The *PHEX* mutations were detected in 6 familial and 3 sporadic hypophosphatemic rickets/osteomalacia. Altogether, 2 novel mutations were detected: 1 missense mutation c.1183G>C in exon 11, resulting in p.Gly395Arg and 1 missense mutation c.1751A>C in exon 17, resulting in p.His584Pro. No mutations were found in the 250 healthy controls.

**Conclusions:**

Our study increases knowledge of the *PHEX* gene mutation types and clinical phenotypes found in Chinese patients with XLH, which is important for understanding the genetic basis of XLH. The molecular diagnosis of a *PHEX* genetic mutation is of great importance for confirming the clinical diagnosis of XLH, conducting genetic counseling, and facilitating prenatal intervention, especially in the case of sporadic patients.

## Introduction

Hypophosphatemic rickets/osteomalacia is characterized by defective renal phosphate re-absorption and abnormal bone mineralization [1]–[4]. Clinical features of the disease include short stature, bone and/or joint pain, lower extremity deformities, calcification of the ligaments, and dental abnormalities. X-linked dominant hypophosphatemia (XLH), autosomal dominant hypophosphatemic rickets (ADHR), and autosomal recessive hypophosphatemic rickets (ARHR) are the three main types of hypophosphatemic rickets and are associated with mutations in the *phosphate-regulating endopeptidase* gene (*PHEX*), the *fibroblast growth factor 23* gene (*FGF23*), and the *dentin matrix acidic phosphoprotein1* gene (*DMP1*), respectively. XLH (MIM 307800), which was first reported in 1939 [5], is the most common genetic form of hypophosphatemic rickets/osteomalacia and has an incidence rate of 3.9–5.0 per 100,000 [6]–[8]. In familial hypophosphatemic rickets (FHR), hypophosphatemic rickets/osteomalacia can be inherited in either an X-linked autosomal dominant or autosomal recessive manner. The most common disease-causing genetic mutations in these cases occur in the *PHEX* gene and cause 87% of familial XLH and 72% of the sporadic cases [9]. XLH is characterized by renal phosphate wasting, which causes hypophosphatemia and normal to low 1,25-dihydroxy-vitamin D3 serum levels [10]; together, these indicate defects in phosphate and vitamin D metabolism.

The *PHEX* gene is located on chromosome Xp22.1, consists of 22 exons, spanning 220 kb with 6172 bp of transcript length, and encodes a membrane-bound metalloprotease composed of 749 amino acids [10], [11]. The PHEX protein shares a common overall structure with other members of the neutral endopeptidase family, including neprilysin, two endothelin-converting enzymes (ECE-1 and -2), the KELL antigen, and the damage-induced neuronal endopeptidase/X-converting enzyme [3], [10]. The structure consists of a short N-terminal intracellular region, a single N-terminal hydrophobic region that corresponds with the transmembrane domain, a highly conserved zinc-binding domain in exons 17 and 19, and a large extracellular C-terminal domain [3], [10]. The PHEX protein is predominantly expressed in cartilage, osteoblasts, and odontoblasts but not in the kidney [10], [12], [13]. Although the exact mechanism of how *PHEX* mutations cause rickets/osteomalacia remains unknown, some studies have shown that PHEX may inactivate bone mineralization inhibitors and that one of the extraosseous consequences of PHEX inactivation includes an increase in the level of FGF-23 [14].

Currently, 329 mutations in the *PHEX* gene have been reported in the *PHEX* mutation database (http://www.phexdb.mcgill.ca; Mar 17 12:35∶21 2014), which mostly occur in European (Northern European), North American, and Far Eastern populations. According to the *PHEX* mutation database, the frequencies of the different types of mutations include the following: 27% frameshifts, 19.8% abnormal splicing, 19.4% missense, 18% nonsense, 28% deletions, and 2.4% polymorphisms. However, only 14 mutations (2 deletion mutation, 3 nonsense mutations, 1 frameshift mutation, 3 donor splice site mutations, and 5 missense mutations) in the *PHEX* gene have been reported in Chinese patients with familial XLH [15]–[20]. In this study, we identified *PHEX* gene mutations in Chinese patients (familial and sporadic) with hypophosphatemic rickets/osteomalacia in order to elucidate the *PHEX* gene mutation types and clinical features observed in Chinese patients.

## Materials and Methods

### Study Subjects

**Figure 1:**
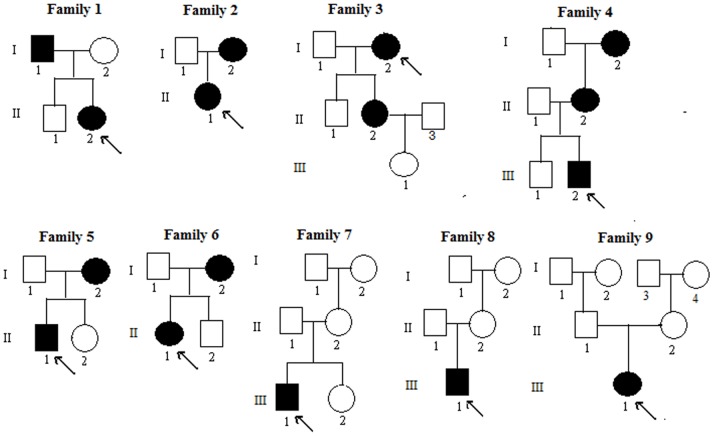
Pedigree of the familial patients with XLH. Black symbols represent the affected individuals, and open symbols represent the unaffected individuals. Circles and squares represent the females and males, respectively. The arrows identify the probands in the families.

The Department of Osteoporosis and Bone Diseases recruited all of the subjects involved in the study over a 6-year period. All of the subjects were of Chinese Han ethnicity and had non-consanguineous parents. Diagnosis of XLH was based on clinical manifestations, radiology results, skeletal deformities, growth impairment, and laboratory results that indicated the occurrence of hypophosphatemia and renal phosphate wasting. Altogether, 45 individuals including 16 patients from 9 unrelated Chinese families were investigated in our study. Three patients were from family 4. Family 7, 8 and 9 had only 1 patient each. The other families (Family 1, 2, 3, 5 and 6) had 2 patients each. The pedigrees of X-linked hypophosphatemic rickets are shown in Figure 1. The fasting blood was collected from each of the subjects during a clinic visit and was analyzed in the central laboratory of Shanghai JiaoTong University Affiliated Sixth People’s Hospital. Serum samples were used to measure calcium (Ca), phosphate (P), parathyroid hormone (PTH) concentrations, alkaline phosphatase (ALP) activity, and the Serum 25-hydroxyvitamin D [25(OH)D] level. Serum concentrations of Ca, P and ALP were measured by using Hitachi 7600 automatic biochemical analyzer. Serum 25(OH)D and PTH were measured by ECLIA Elecsys autoanalyzer (E170; Roche Diagnostic GmbH, Mannheim, Germany). The intra-assay coefficients of variation (CVs) for 25(OH)D were 5.7% at a level of 25.2 ng/mL, 5.7% at a level of 39.9 ng/mL and 5.4% at a level of 65.6 ng/mL, respectively. The inter-assay CVs for 25(OH)D were 9.9% at a level of 25.2 ng/mL, 7.3% at a level of 39.9 ng/mL and 6.9% at a level of 65.6 ng/mL, respectively. The intra-assay and inter-assay CVs were 1.4% and 2.9%, respectively, for PTH. All of the laboratory data were collected prior to treating the patients with surgery or medicine.

### Ethics Statement

The Ethics Committee of the Shanghai JiaoTong University Affiliated Sixth People’s Hospital approved this study and the project was conducted following the terms of “Declaration of Helsinki”. Signatures confirming informed consent were obtained from the participating subjects before starting the project. In addition, we obtained written informed consent from the parents on the behalf of the minor/children participants involved in our study.

### Mutation Analysis

Genomic DNA was isolated from peripheral blood leukocytes using the conventional phenol-chloroform extraction method. We screened the *PHEX* gene completely for mutations in 16 patients, the other phenotype normal family members, and 250 healthy ethnically matched controls (125 males and 125 females, Chinese Han ethnicity). The DNA sequence for the *PHEX* gene was obtained from available online database (NCBI Reference Sequence Accession Number: NG_007563.2; the mRNA Accession Number: NM_000444.5.). The PCR and sequencing primers were the same as we used in our previous study [18]. All 22 exons and exon-intron boundaries in the *PHEX* gene were amplified by polymerase chain reaction (PCR). Hot Start PCR reaction was performed in our study, and HotStar Taq DNA polymerase (Qiagen Inc.) was used for highly specific amplification in hot-start PCR reaction. The cycling program of amplification was 95°C for 15 minutes; 11 cycles of 94°C for 15 seconds, 62°C per cycle for 40 seconds, 72°C for 1 minutes; 24 cycles of 94°C for 15 seconds, 57°C for 30 seconds, 72°C for 1 minute, 72°C for 2 minutes. Direct sequencing was performed using the BigDye Terminator Cycle Sequencing Ready Reaction Kit, version 3.1 (Applied Biosystems, Foster, CA, USA), and the cycling program of sequencing was 96°C for 1 minute; 28 cycles of 96°C for 10 seconds, 50°C for 5 seconds, 60°C for 4 minutes. The resulting PCR products were directly sequenced using an automated ABI PRISM 3130 sequencer (Foster, CA). Meanwhile, once a mutation was detected, we performed PCR amplification in the same DNA sample again by using HotStar HiFidelity polymerase (Qiagen Inc.) for highly sensitive and reliable high-fidelity hot-start PCR. Then, the purified PCR product was sequenced from the other strand to further verify the mutation. Single-nucleotide polymorphisms (SNPs) were identified using Polyphred (http://droog.mbt.washington.edu/poly_get.html). Novel mutations were identified using HGMD (http://www.hgmd.cf.ac.uk/). Mutations were confirmed using Mutalyzer 2.0 (http://mutalyzer.nl/check). The DNA sequences obtained were aligned with homologous sequences that had been deposited into GenBank using the CluxtalX 1.83 algorithm [16].

### Mutation Prediction

Polyphen-2 [19] and Sorting Intolerant from Tolerant (SIFT) were used to determine the functional effects of all the missense mutations in the *PHEX* gene [8]. Polyphen-2 (http://genetics.bwh.Harvard.edu/pph2/) and SIFT (http://sift.jcvi.Org/www/SIFT_enst_submit) are tools that predict the possible impacts of an amino acid substitution on the structure and function of a human protein using a straightforward physical comparative analysis [21]–[24]. For Polyphen-2, the following three empirically derived outcomes were used: most likely damaging (with a high confidence level, the substitution will affect protein function or structure), possibly damaging (the substitution is supposed to affect protein function or structure), and benign (the substitution most likely lacks any phenotypic effect). The SIFT score [8], [25] represents the normalized probability that the amino acid change is tolerated. The SIFT score <0.05 are predicted to be deleterious.

## Results

### Clinical Features of the Subjects

**Figure 2:**
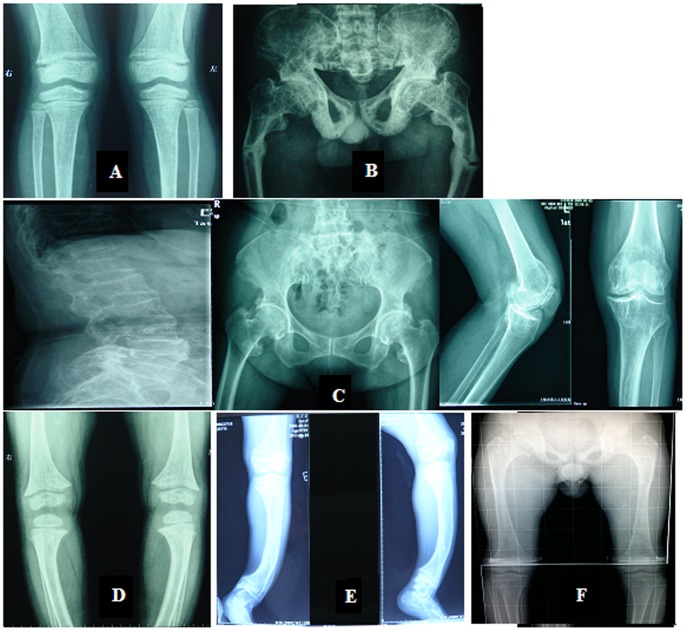
Radiology results for the patients. (A): A general decrease in bone density, widened metaphyses, bilateral femoral distal metaphysis epiphyseal line blurred, and similar features in the lower tibia are shown. (B): A high bone mineral density, genu varum deformities in the lower limbs, and a Looser Zone in the bilateral femoral shaft are shown. (C): A general decrease in the bone density of the vertebrae and pelvis, bilateral hip and knee joint degeneration, and multiple vertebral wedge changes are shown. (D): Widened metaphyses is shown. (E): A high bone mineral density and anterior bowing of the lower limbs are shown. (F): A general decrease in the bone density of the vertebrae and pelvis, an enlargement of the epiphyseal and metaphyseal portions of the under section of the femoral, a short and wide femoral neck, and a large capital femoral epiphyses are shown.

The general features and laboratory results of patients are shown in Table 1. The radiology results of the patients are shown in Figure 2. The phenotypes and laboratory results for the family members except patients were all normal (data not shown).

**Table 1:** General features and laboratory results for the XLH patients studied.

Family No.	Patient No./Gender	Age (year)	Height (cm)	Weight (kg)	BMI (kg/m^2^)	Height percentile	Ca (mmol/l)	P (mmol/l)	ALP (u/l)	PTH (u/l)	25(OH)D (ng/ml)
1	II2/F	5	92	13.5	**15.95**	**<1st**	2.35	**0.73**	**475**	**78.56**	20.20
1	I1/M	34	147	54	24.99	**<5th**	2.30	**0.68**	**168**	49.80	**15.30**
2	II1/F	4	95	15	**16.62**	**<3rd**	2.43	0.98	**752**	**83.04**	**6.78**
2	I2/F	27	139	45	23.29	**<5th**	2.28	**0.54**	45	**67.08**	**5.63**
3	II2/F	43	150	51	22.67	**<25th**	**2.67**	**0.63**	**121**	55.14	**19.70**
3	I2/F	73	145	53	25.21	**<25th**	2.36	**0.63**	**157**	**132.10**	**<4**
4	III2/M	4	99	17	**17.35**	**<15th**	2.38	**0.74**	**507**	50.62	**17.99**
4	II2/F	33	141	41	20.62	**<5th**	2.17	0.83	66	**71.21**	**9.66**
4	I2/F	58	127	48	29.76	**<5th**	2.26	0.91	**168**	**67.52**	**18.77**
5	II1/M	14	137	31.5	**16.78**	**<1st**	2.32	**0.71**	**409**	61.25	**<4**
5	I2/F	37	137.5	47	24.86	**<5th**	2.18	**0.70**	79	**77.62**	**<4**
6	II1/F	13	142	45	22.32	**<3rd**	2.34	**0.67**	**122**	**84.52**	**13.08**
6	I2/F	37	138	41	21.53	**<5th**	2.19	**0.70**	109	**101.80**	**17.48**
7	III1/M	16	145	40.5	19.26	**<1st**	2.25	0.85	**534**	50.12	21.23
8	III1/M	3	82	12	**17.85**	**<1st**	2.32	0.89	**414**	**87.41**	27.36
9	III1/F	11	98	21	21.87	**<1st**	2.22	**0.65**	**359**	**107.63**	**5.38**

Footnotes: Abnormal data are bolded. The normal range for phosphate is 0.8–1.6 mmol/l; for calcium is 2.08–2.60 mmol/l; for alkaline phosphatase (ALP) is 15–112 u/l; for parathyroid hormone (PTH) is 15–65 µ/l; and for 25-OH vitamin D [25(OH)D] is 20–35 ng/ml. F: female, M: male. BMI (Body Mass Index) is defined as the individual’s body mass divided by the square of their height. BMI normal range is 18.5–25 Kg/m^2^. The data of height percentile referenced the standard provided by the World Health Organization.

In family 1, the proband (II2, 5 years) had a short stature (92 cm tall) with an abnormal gait and retarded dentition, whose imaging results are shown in Figure 2A. While her father (I1, 34 years) even had more severe phenotypes, besides a short stature (147 cm height) and an abnormal gait. His onset age was 1.5 years and had genu varum with an “O” appearance. In addition, he suffered a bilateral femoral fracture at 7 years of age and the imaging results are shown in Figure 2B. In family 2, the proband (II1, 4 years) was characterized by delayed dentition and genu varum. While her mother (I2) had more severe phenotypes with earlier onset age (1.5 years), consisted of an abnormal gait, delayed dentition, odontodysplasia, and genu varum with an “O” appearance. The symptoms were even worse as aging. Her teeth started falling out at 22 years of age, and only 5 teeth remained currently. In family 3, the proband’s (I2, 73 years) clinical features consisted of an abnormal gait, kyphosis, and hip and knee joint pain. The X-ray results are shown in Figure 2C. However, her daughter (II2, 43 years) only had mild genu varum and bone pain. In family 4, the proband (III2, 4 years) had short stature and mild genu varum. The X-ray results are shown in Figure 2D. His mother (II2, 33 years) and grandmother (I2, 58 years) had the same symptoms as he. In family 5, the proband (II1, 14 years) was delivered by Caesarean section and had a low birth weight of 2.45 kg. The proband had obvious growth retardation and stopped growing after 9 years of age. He also suffered from hip and knee joint pain. His mother’s (I2, 37 years) first clinical abnormalities were detected at 4 years of age and consisted of an abnormal gait and growth retardation. Genu varum with an “O” appearance developed as aging, and her height stopped growing at 16 years of age after the onset of her menstrual cycle. Her teeth began to fall out at 17 years of age, and only 1 tooth remained at present. In family 6, the proband (II1, 13 years) was characterized by goyectyposis. The X-ray results are shown in Figure 2E. Her mother (I2, 37 years) had the similar symptoms as her daughter. The first sporadic patient (III1, 16 years) was from family 7. When he was 9 months old, he experienced swelling of his hip joints and was diagnosed with rickets when he was 11 months old. He was characterized by genu varum, hip pain and growth retardation. Imaging results are shown in Figure 2F. The second sporadic patient (III1, 3 years) was from family 8. When he began to walk at 1.5 years of age, genu varum was detected. The X-ray results revealed cephalus quadratus, bilateral femur bending, and mild bilateral genu varum in the knees (data not shown). The third sporadic patient (III1, 11 years) was from family 9. Her first clinical abnormality was detected when she was 16 months old and characterized by an abnormal gait (genu varum with “O” appearance).

### Mutation Analysis of the *PHEX* Gene

The mutational analysis of the *PHEX* gene in our patients revealed the following **2** novel mutations and they both were detected in family 2∶1 missense mutation, c.1183G>C, in exon 11, resulting in p.Gly395Arg and 1 missense mutation, c.1751A>C in exon 17, resulting in p.H584P. The main clinical results from the genetic testing of the patients at the time of this study are summarized in Table 2.

**Table 2:** Summary of the patient clinical data.

Family No.	Patient	Gender/Age of onset (year)	Clinical findings	Mutation site	*PHEX* mutation	Inheritance
1	(II-2) daughter	F/3	Retarded dentition	Exon 20	Trp660X (c.1980G>A)	Familial
1	(I-1) father	M/1.5	Genu varum	Exon 20	Trp660X (c.1980G>A)	Familial
2	(II-1) daughter	F/2	Genu varum; retarded dentition	**Exon 17**	**His584Pro (c.1751A>C)**	Familial
2	(I-2) mother	F/1.5	Genu varum; retarded dentition; odontodysplasia; teeth falling out	**Exon 17** **Exon 11**	**His584Pro (c.1751A>C)** **Gly395Arg (c.1183G>C)**	Familial
3	(II-2) daughter	F/4	Genu varum and bone pain	Exon 12	Trp444X (c.1332 G>A)	Familial
3	(I-2) mother	F/5	Hip and knee joint pain; kyphosis; bone pain	Exon 12	Trp444X (c.1332 G>A)	Familial
4	(III2) son	M/1.5	Genu varum	Intron 15	c.1646-2A>T	Familial
4	(II2) mother	F/2	Genu varum	Intron 15	c.1646-2A>T	Familial
4	(I2) grandmother	F/5	Genu varum	Intron 15	c.1646-2A>T	Familial
5	(II1) son	M/5	Genu varum (mild); bone pain; growth retardation	Intron 10	c.1174-1G>A	Familial
5	(I2) mother	F/4	Genu varum; odontodysplasia; teeth falling out; growth retardation	Intron 10	c.1174-1G>A	Familial
6	(II1) daughter	F/2	Bowing of legs	Exon 16	Tyr565Phefsx5 (c.1694delA)	Familial
6	(I2) mother	F/2	Bowing of legs	Exon 16	Tyr565Phefsx5 (c.1694delA)	Familial
7	(III1) son	M/0.75	Genu varum; hip pain; growth retardation	Intron 17	c.1768+2T>G	Sporadic
8	(III1) son	M/1.5	Genu varum; cephalus quadratus	Exon 21	Trp692IlefsX2 (c.2077_*4delinsA)	Sporadic
9	(III1) daughter	F/1.3	Genu varum	Exon 15	Arg549X (c.1645C>T)	Sporadic

Footnotes: Novel mutations are bolded.

**Figure 3:**
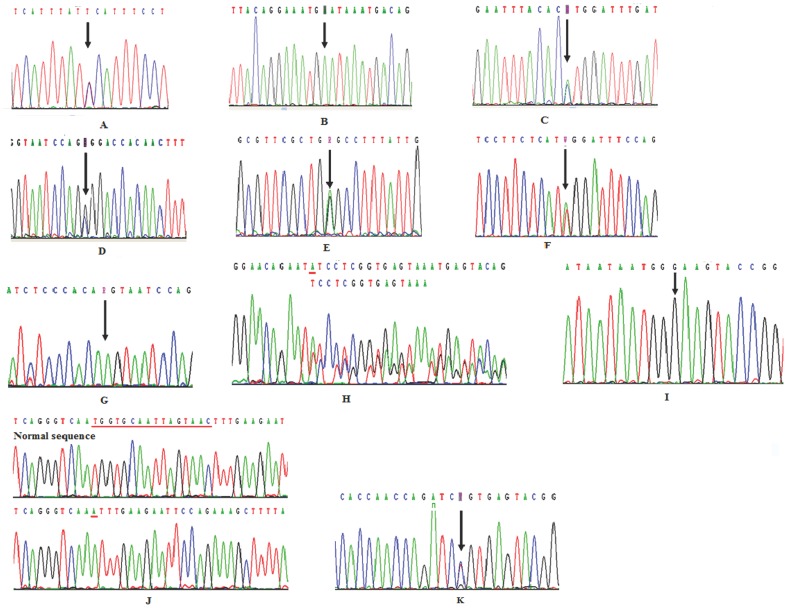
*PHEX* gene mutation sequencing diagram. (A): A nonsense mutation c.1980G>A in exon 20 was detected in Proband (II-2) from family 1. (B): Proband’s father (I-1) was hemizygous for a nonsense mutation, c.1980G>A in exon 20 from family 1. (C): A novel missense mutation, c.1751A>C in exon 17, was identified in proband (IV-1) and her mother (III-2) from family 2. (D): A novel missense mutation, c.1183G>C in exon 11, was identified in III-2 from family 2. (E): A nonsense mutation, c.1332G>A in exon 12, was identified in proband (I-2) and her daughter (II-2) from family 3. (F): A putative aberrant splicing mutation, c.1646-2A>T in intron 15, was identified in proband (III 2), his mother (II2), and his grandmother (I2) from family 4. (G): A putative aberrant splicing mutation, c.1174-1G>A in intron 10, was identified in proband (II1) and his mother (I2) from family 5. (H): A deletion mutation, c.1694delA in exon 16, was identified in proband (II1) and her mother (I2) from family 6. (I): A splicing mutation, c.1768+2T>G in intron 17, was identified in proband (III1) from family 7. (J): A del-insertion mutation, c.2077_*4delinsAin exon 21, was identified in proband (III1) from family 8. (K): A nonsense mutation, c.1645C>T in exon 15, was identified in proband (III1) from family 9.

In family 1, the clinical diagnosis of XLH in the proband (II2) and her father (I1) was confirmed by the detection of a nonsense mutation in codon 660 in exon 20 of the *PHEX* gene (c.1980G>A), which results in the replacement of a tryptophan residue with a premature stop codon (p.Trp660X). (The sequences of II2 and I1 were shown in figure 3A and 3B, respectively). In family 2, a novel missense mutation was detected in the proband (II1) and her mother (I2) in which a proline is substituted for a histidine at position 584 as a result of a mutation in exon 17 of the *PHEX* gene (c.1751A>C; p.His584Pro; Figure 3C). Interestingly, the mother was harbouring another novel missense mutation in codon 395 in exon 11 of the *PHEX* gene (c.1183G>C), which results in an arginine replacing a glycine (p.Gly395Arg; Figure 3D). In family 3, sequence analysis of the proband (I2) and her daughter (II2) revealed a nonsense mutation in codon 444 in exon 12 of the *PHEX* gene (c.1332G>A), which results in a premature stop codon replacing a tryptophan residue (p.Trp444X; Figure 3E). In family 4, the proband (III2), his mother (II2), and his grandmother (I2) carried a putative aberrant splicing mutation c.1646-2A>T in intron 15 at splicing acceptor sites (Figure 3F). In family 5, the proband (II1) and his mother (I2) carried a putative aberrant splicing mutation c.1174-1G>A in intron 10 at splicing acceptor sites (Figure 3G). In family 6, the proband (II1) and her mother (I2) carried a heterozygous deletion of one nucleotide (A) in codon 565 (c.1694delA), which causes a phenylalanine to be substituted for a tyrosine at position 565 and replaces the next 5 amino acids with a stop codon (exon 16 of the *PHEX* gene; c.1694delA; p.Tyr565Phefsx5; Figure 3H).

In the 3 sporadic cases (family 7, 8, and 9), the proband (III1) from family 7 carried a splicing mutation c.1768+2T>G in intron 17 (Figure 3I); the proband (III1) from family 8 carried a deletion mutation c.2077_*4delinsA in exon 21 that results in p.Trp692IlefsX2 (Figure 3J); and the proband (III1) from family 9 carried a nonsense mutation c.1645C>T in exon 15 that results in p.Arg549X (Figure 3K).

No mutation was detected in the phenotype normal family members and 250 ethnically matched control individuals.

**Figure 4:**
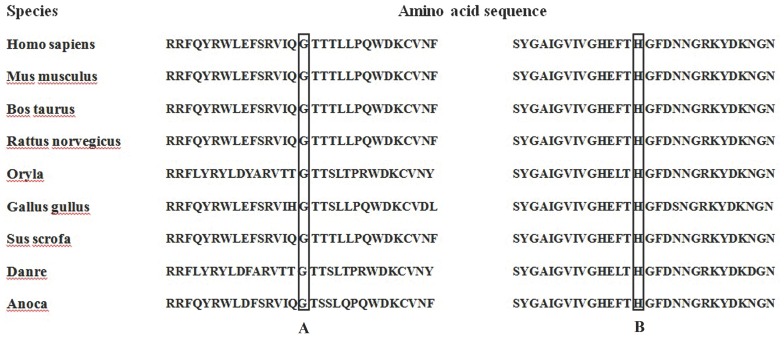
Partial amino acid sequences of the *PHEX* gene from 9 species. The amino acids at p.395 in exon 11 (A) and p.584 in exon 17 (B) are highly conserved in 9 different species.

To evaluate the consequence of the p.Gly395Arg and p.His584Pro mutations, PolyPhen-2 and SIFT analyses of the mutations were performed. Both mutations were predicted to be most likely damaging (PolyPhen-2 score of 0.98 and 0.99, respectively. Both SIFT score <0.05). Meanwhile, the aminoacid residues at p.395 and p.584 are highly conserved across 9 different biological species (Figure 4A and 4B).

## Discussion

In this study, we identified 10 different *PHEX* mutations (including **2** novel mutations) in 16 patients from 9 unrelated families (probands from family 7, 8, and 9 are sporadic patients) with XLH and reported the different clinical features observed in these Chinese patients.

The nonsense mutations p.Trp660X in exon 20, p.Trp444X in exon 12, and p.Arg549X in exon 15 may result in the translation of truncated proteins that lack exons 20 to 22, exons 12 to 22, and exons 15 to 22, respectively. Four cysteine residues are located within this C-terminal region and are highly conserved in the PHEX protein [3], [10]. These 4 cysteine residues are most likely involved in disulfide bond formation, and losing them could result in a defective secondary protein structure that could greatly inhibit the enzymatic activity of the protein. Therefore, of all of the mutations detected in this study, these 3 mutations are the most likely to affect the function of the PHEX protein. It is known that approximately 27% of the mutations in the *PHEX* gene are nonsense mutations [26]. After searching the *PHEX* mutation database, 15 mutations were detected in exon 20 [18], indicating that exon 20 may be a mutational hotspot.

Two novel missense mutations were detected in family 2: p.His584Pro in exon 17 and p.Gly395Arg in exon 11. The *PHEX* gene contains 10 highly conserved cysteine residues, all of which are located in a very large extracellular domain. These cysteine residues may be involved in disulfide bond formation and protein folding [24]. The p.His584Pro and p.Gly395Arg mutations affect 2 of these cysteine residues. Mutations at both sites would most likely result in changes to the protein structure and would result in the loss of protein function. Additionally, glycine (G) and proline (P) are non-polar hydrophobic amino acids, and arginine (R) and histidine (H) are polar alkaline hydrophilic amino acids. Therefore, it is predicted that substituting G with R and H with P will alter the biochemical properties at these positions. Furthermore, p.His584Pro and p.Gly395Arg are non-conservative, affect evolutionarily highly conserved amino acids from nine different species (Fig. 4) and were predicted in silico by all bioinformatic tools used to be of pathogenic relevance (PolyPhen-2 score of 0.98 and 0.99, respectively. Both SIFT score <0.05). The proband’s mother (I2) in family 2 carried both mutations (p.His584Pro and p.Gly395Arg), therefore has more severe phenotypes than her daughter. These phenotypic symptoms included a lower blood phosphorus level, an earlier onset age, odontodysplasia, delayed dentition, and teeth falling out at the age of 22 years.

Two deletion mutations were identified in our study. One deletion mutation was p.Tyr565Phefsx5 in exon 16. This mutation consists of a heterozygous deletion of one nucleotide (A) in codon 565 (c.1694delA), which leads to a phenylalanine substitution for a tyrosine at P.565 and a subsequent premature truncation at p.570, which results in a nonfunctioning PHEX product. The other mutation is an insertion-deletion mutation, c.2077_*4delinsA. In this mutation, 16 nucleotides (TGGTGCAATTAGTAAC) from codon 718 to codon 723 are deleted and 1 nucleotide (A) is inserted at p.N718, which results in 6 amino acids missing from p.N718 to p.N723 and a lysine insertion (at codon 718). These changes most likely alter the biochemical properties at these positions and affect the PHEX protein function.

Three splice site mutations were identified in our study. Splice site mutations at introns 10, 15, and 17 (IVS10-1G>A, IVS15-2A>T, and IVS17+2T>G, respectively) were predicted to cause exons 11, 16, and 18 to be skipped, respectively. These changes would result in a reading frame shift and truncated proteins.

The *PHEX* gene mutations in the 3 sporadic patients (probands from family 7, 8, and 9) were not inherited from their parents and are most likely *de novo* mutations. These types of mutations are caused by a mutation in the germ cell or germ cell mosaicism in the gonads of one of the parents or by a mutation in the fertilized egg itself. Studies have shown that male mutation bias frequently occurs among higher organisms, and in humans, the male to female bias ratio is approximately 6 to 1 because of differences in male and female gamete formation [24], [27]. Furthermore, the male germline accumulates more DNA replication errors because of the higher number of germline cell divisions in males than females [28]. Therefore, *PHEX* mutagenesis in paternal germ cells is likely more frequent in sporadic patients and would only affect the female offspring [24], [29], which is accordance with our finding from family 9 (proband III1 is female). Interestingly, however, that the 2 sporadic patients (probands from family 7 and 8) in our study are males, which differs from the demographics in previous studies. This finding indicates that the mutated *PHEX* alleles in sporadic male patients probably resulted from the mutagenesis in the X chromosome of the maternal germ cell.

From our study, there are no significant differences of gene mutation types and mutation locations in the *PHEX* gene in Chinese XLH patients compare to non-Chinese patients. However, the same mutations in different races can cause different clinical features. For example, p.Trp444X was firstly reported by Beck-Nielsen SS, et al. [30] in a sporadic patient, a Danish male, with a normal height, mild skeletal and endodontic phenotype. Whereas, in our study, the mutation was found in familial patients with abnormal gait, kyphosis, and hip and knee joint pain. In addition, we identified that the proband and her daughter carried the non-sense mutation (p.Trp444X) which consisted of a heterozygous G to A transition at c.1332 in exon 12, while, The mutation reported by Beck-Nielsen SS, et al. is c.1331G>A affecting one nucleotide upstream the one described in our manuscript (c.1332G>A). Although, the result is the same at the protein level with a tryptophan at position 444 being substituted by a stop codon and truncation at p. 444, the clinical features are quite different in Chinese patients compare to non-Chinese patients.

Although, no evident genotype phenotype correlation could be established in our study, 2 novel mutations were detected and different clinical features were described. Therefore, Functional studies investigating the *PHEX* gene mutation should be performed to elucidate the complex relationship between genotype and phenotype.
